# The Effect of Sociocultural Attitudes on Developing Eating Disorders Among Young Females in Almadinah Almunawarah, Saudi Arabia

**DOI:** 10.7759/cureus.50576

**Published:** 2023-12-15

**Authors:** Nadir Makki, Shatha A Althubyani, Rose Q Mobarki, Joud A Alsayed, Roaa J Almohammadi, Razan A Baabdullah

**Affiliations:** 1 Psychiatry, Taibah University, Madinah, SAU; 2 Psychiatry and Behavioral Sciences, Taibah University, Madinah, SAU; 3 Medicine and Surgery, Taibah University, Madinah, SAU; 4 College of Medicine, Taibah University, Madinah, SAU

**Keywords:** body image, sociocultural attitudes, ed risk, eating attitudes, eating disorders

## Abstract

Background: Eating disorders (ED) are believed to be more susceptible in women due to varied factors involving dissatisfaction with their body and appearance. The exact cause of ED isn't known. But it may be triggered by biological, psychological, environmental, and social factors.

Objectives: The current literature aims to explore the body dissatisfaction of women from Almadinah Almunawarah and factors that may contribute to developing risk of ED and assess the discrepancies between desired and healthy BMI.

Methods: The Sociocultural Attitudes Toward Appearance Questionnaire-4 (SATAQ-4) questionnaire surveyed 384 females to explore family, peer, and media pressure, followed by the Eating Attitudes Test-26 (EAT-26) questionnaire to recognize those at risk of developing ED. The body dissatisfaction of the sample was measured by the difference between the healthy BMI and the desired BMI.

Results: A total of 127 of the participants, who were reported to have a high probability of developing an ED, had the highest factor scored in the SATAQ-4 questionnaire being media exposure with a p-value less than 0.001. The study showed a difference in the ideal body and what is considered a healthy BMI. Results showed no correlation between BMI and developing ED.

Discussion: Women of younger age groups are more vulnerable to being under the influence of sociocultural attitudes, thus they are more susceptible to developing risky eating behaviors. This can be affected by family, peers, and media factors.

Conclusion: The findings of this study show a high prevalence of risky eating behaviors, particularly among those who experience family and media pressure toward body shape and weight. Peer pressure was also identified as a significant risk factor. These findings emphasize the need for interventions that target sociocultural attitudes and provide support for vulnerable individuals.

## Introduction

Eating disorders (ED) involve an excessive concern regarding one’s diet, appearance, weight and body shape. This obsession can be nearly fatal in some cases as it may cause an extreme change in dietary and exercise habits that can harm one’s physical and mental well-being and decrease quality of life. Anorexia nervosa, bulimia nervosa, and binge-eating disorder are considered common ED [1].

It is still unclear what causes ED exactly. However, it's theorized to be related to anxiety and depression [1]. The general strain theory has been used to explain how negative emotions can influence behavior; some exhibited outwardly and others internalized the negativity that could manifest as anxiety, depression and disordered eating behavior [1]. It’s also believed to be occurring more often in women as many studies have found a higher incidence of ED in women [2], triggered by biological, psychological, environmental and social factors [3].

Psychological factors like depression, anxiety and body-image dissatisfaction can alter self-perception, and lead one to pursue disordered eating behaviors as a result [1].

Environmental and sociocultural factors such as negative parental feedback regarding their child’s appearance, a critical family environment and comparing one’s appearance to those displayed in media and accepted by society at large and closer relations (family, friends, peers, etc.) as ideals, those of which usually apply to the popularized standard of thinness as the desirable female beauty ideal can also lead to developing risky eating behaviors in order to achieve that “ideal” image [1,2].

Sociocultural attitudes regarding, appearance and body image represent the internalization of self-value by identifying the standards of sociocultural beauty through societal, familial, peer and mass media influence and the perception of these individuals towards their body image is affected by many variables [4,5].

Interaction with family members tends to be the first source of one’s integration with society, which often influences self-esteem and perception of one’s body image and possibly adopting disturbed attitudes toward their eating and exercising habits that may affect their health [6].

While growing up surrounded by peers who have their own ideas regarding their body image and possibly some extreme dieting and exercising habits some people may be influenced or feel pressured to fall into those ideas and perceive them as the norm, which further disturbs one’s relationship with their body [7].

Another important factor related to the high likelihood of developing ED in young women is the perceived ideal standards of beauty and body shape promoted through mass media [7]. Studies have shown that the sociocultural influence of Western media might have brought on the newly established standards of beauty and body shape in Eastern societies like the Middle East which has increased body dissatisfaction and poor eating habits in young females [2].

The correlation between sociocultural attitudes including parental factors, mass media, body image apprehensions resulting from peer pressure and the onset of ED among females isn’t often discussed in literature, especially in our region and for that reason, our aim is to explore how can society and socially imposed standards of appearance and beauty and the internalization and acceptance of these standards by young women affect and possibly increase the probability of developing ED in young women in Almadinah Almunawarah.

This study’s objectives are to explore the concerns regarding body image and unhealthy eating behaviors in young women in Almadinah Almunawarah, to identify the important social and cultural factors contributing to the development of ED, to review the discrepancies between idealized standards of body weight in society and normal BMI (18.5 to 24.9), to assess the risky behaviors suggestive of a higher possibility to develop an ED and to discuss how sociocultural attitudes towards appearance can have a role in developing ED in young women in Almadinah Almunawarah.

## Materials and methods

Study setting and population

A cross-sectional study was conducted between April 2022 and May 2023. The targeted population was young females living in Almadinah Almunawarah, Saudi Arabia. A total of 384 women aged 18-30 years participated in this study. The criteria for inclusion in our study comprised young women aged 18 to 30 residing in Almadinah. Whereas, the exclusion criteria encompassed males, individuals in prison, pregnant women, females below 18 or above 30 years old, and those with cognitive or mental disabilities.

Ethical statement

The study’s protocol and questionnaires were approved by the Research Ethics Committee, College of Medicine, Taibah University. Ethical approval was obtained in January 2022. Study ID is STU-21-027 (Reference Number: IORG0008716-IRB00010413), in Almadinah Almunawarah, Saudi Arabia. Participants' confidentiality and anonymity were assured. Consent was obtained from those who agreed to participate.

This study’s sample was determined using the Krejcie & Morgan equation for sample size calculation that minimized standard error. The population census of women in Almadinah Almunawarah in 2017 was 239,234. According to Krejcie & Morgan (1970), the required sample size for a population (N>= 100000) is 384 participants at a confidence interval (CI) of 95%. And the study has 384 participants.

Data collection tools

Using a web-based survey, data was collected anonymously via a secure server using Google Forms. The link https://forms.gle/FZpVjKaSWpRxEcFm8 was distributed among participants on-site (Gym, Schools, Malls, etc.) and through social media platforms. The participants responded to the survey after giving their consent regarding obtaining personal data, including age, weight, and height. Body mass index (BMI) was calculated using self-reported measures of height and weight submitted by participants. Using the World Health Organization's cutoff criteria for BMI, participants were classified into underweight, normal weight, overweight and obese. Validated questionnaires were used in the survey and translated from original English into the Arabic language using a certified translator.

Questionnaires Included the Following Validated Scales

The Sociocultural Attitudes Toward Appearance Questionnaire-4 (SATAQ-4): SATAQ-4 is a 22-item questionnaire collecting data related to social pressure faced by media, family and peers potentially affecting the prevalence of eating disorders, each question is measured on a 1-5 scale. The questionnaire explores four sociocultural domains: self-pressure to be “thin or muscular”; family pressure (parents, siblings, close and distant relatives); peer pressure (friends, colleagues, and other social relations); and media pressures (internet and social media, movies and television, advertisements) regarding weight and appearance [8].

The Eating Attitudes Test (EAT-26): EAT-26© is used for recognizing people at risk of developing disordered eating behaviors. The EAT-26 test was proven to be effective and is used widely across various countries and ages. It is a self-reported assessment tool with answers ranked on a 1-6 point scale. When the overall score was reported at 20 points or more, participants were classified to be at risk of developing disordered eating attitudes and habits. Additional assessment by a mental health expert is advised for those with high scores [9].

Statistical analysis

Upon collecting the data through a survey and converting it to an Excel sheet, we used the Statistical Package for the Social Sciences (IBM SPSS Statistics for Windows, IBM Corp., Version 26.0, Armonk, NY) for data analysis. Mean and standard deviation (±SD) were used to represent continuous data, while categorical variables were represented as frequency and percentages. Data normality was tested using the Kolmogorov-Smirnov test. Consequently, the Mann-Whitney U test was used in order to determine the relation between the SATAQ-4 subscale and the risk of ED. The Wilcoxon signed ranks test was used to review the discrepancies between idealized standards of body weight in society and the normal BMI. The Spearman’s rank correlation was used to determine the link between disordered eating risk (measured with EAT-26) and BMI. Categorical variable analysis was done using Pearson's Chi-squared o-test to assess the significant difference between the age of the participants regarding intake of eating attitudes test-EAT-26. A multiple logistic regression model was constructed to predict factors affecting ED risk as its dependent binary variable. To verify the assumption of logistic regression, the data were examined to be certain they were not correlated. A p-value less than 0.05 was considered statistically significant, and the CI was 95%.

## Results

The total number of participants was 384, all of them females. In Table 1, which reflects the sociodemographic characteristics of our sample. Approximately 57% (n= 218) of the participants were from 18 to 22 years old. The average height of the participants was 158.4 (SD± 6.2), and their average weight was 58 kg (SD± 14.4). Also, half of the sample has normal weight. And approximately 33.1% (n=127) of the participants scored 20 or more on the EAT-26 scale and they were seen as being susceptible to ED.

**Table 1:** Sociodemographic characteristics of participants and eating disorders risk (N=384).

Characteristics	Frequency	Percentages
Age		
18-22	218	56.8%
23-26	100	26%
27-30	66	17.2%
Body mass index (BMI)		
Underweight	76	19.8%
Normal weight	190	49.5%
Overweight	74	19.3%
Obese	44	11.5%
Eating disorders (ED) risk		
At risk	127	33.1%
Not at risk	257	66.9%

**Figure 1:**
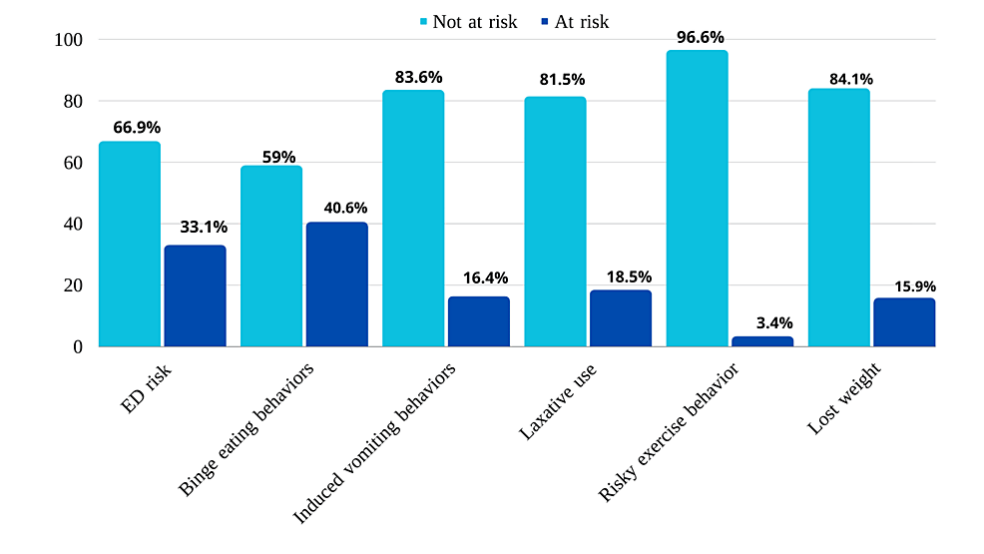
Distribution of participants in the Eating Attitudes Test (EAT)-26 ED = Eating disorders

Figure 1 illustrates the participants' distribution on the EAT-26, the highest prevalence of risk was in binge eating behavior, as around 40.6% (n=156) of the participants were at risk of it. The least prevalence of risk was in risky exercise behavior at 3.4% (n=13).

**Figure 2:**
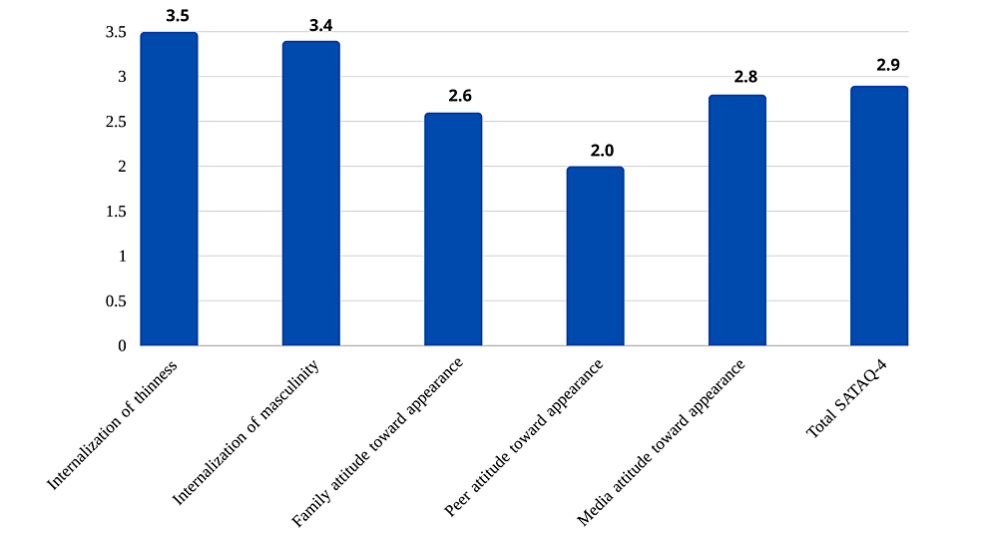
The average response of the Sociocultural Attitudes Toward Appearance Questionnaire-4 (SATAQ-4) for five subscales

The participants' responses and means for each statement in the SATAQ-4 for the whole study sample (n = 384) show a high average of participants' responses, scoring between 3.4 and 3.5 out of 5 for self-pressure to internalization of masculinity, and internalization of thinness, respectively. And the low average of participants (ranging between 2.0 and 2.8 out of 5) agreed with describing family pressure (2.6), peer pressure (2.0), and media pressure (2.8) subscales. The average response on the total score of SATAQ-4 was 2.9 out of 5 (Figure 2).

The participants' response to ED risk part 1 of the EAT-26 shows statements from 1 to 25. Options "sometimes, rarely, and never" were the highest answers the most chosen by participants. While statement 26 “enjoy trying new rich foods" options "always, usually, and often" were the highest answers the most chosen by participants.

Tables 2, 3 show participants' responses to ED risk part 2 of the EAT-26. As shown in the table, for the behavior question “Gone on eating binges where you feel that you may not be able to stop” the “never” option was the most answer chosen by 33.3% (n=128) of participants, and the next option was “once a month or less” option by 26% (n=100), while "once a day or more" was the least option chosen 3.9% (n=15). In behavioral questions "Ever made yourself sick (vomited) to control your weight or shape" and "Ever used laxatives, diet pills, or diuretics (water pills) to control your weight or shape" the "never" option was the most answered chosen by participants 83.6% (n= 321) and 81.5% (n=313) respectively. While about 3.4% (n=13) of participants answered they exercised more than 60 minutes once a day or more. Finally, 15.9% (n=61) of participants reported they lost 20 pounds or more in the past six months.

**Table 2:** Participants' responses to eating disorders risk part 2 of Eating Attitudes Test (EAT)-26 (N=384) (1 out of 2)

Behavioral Questions	Never		Once a Month or Less		2-3 Times a Month		Once a Week			2-6 Times a Week		Once a Day or More		
Gone on eating binges where you feel that you may not be able to stop	128	33.3%	100	26.0%	70	18.2%	46	12.0%		25	6.5%	15		3.9%
Ever made yourself sick (vomited) to control your weight or shape	321	83.6%	19	4.9%	14	3.6%	13	3.4%		7	1.8%	10		2.6%
Ever used laxatives, diet pills or diuretics (water pills) to control your weight or shape	313	81.5%	20	5.2%	13	3.4%	14	3.6%		10	2.6%	14		3.6%
Exercised more than 60 minutes a day to lose or to control your weight	181	47.1%	66	17.2%	48	12.5%	36	9.4%		40	10.4%	13		3.4%

**Table 3:** Participants' responses to eating disorders risk part 2 of Eating Attitudes Test (EAT-26) (N=384) (2 out of 2)

Lost 20 pounds or more in the past 6 months			Yes						No					
			61			15.9%			323				84.1%	

Table 4 shows the difference in mean ranks of ED risk in relation to SATAQ‑4. As shown in the table, internalization to thinness, internalization to masculinity, family pressure, peer pressure, and media pressure, and total scores on SATAQ-4 were all considerably greater among those at-risk participants (P < 0.05). Furthermore, internalization of thinness showed a greater magnitude difference between females at risk (mean rank = 249.2, n= 127) and females who were not (mean rank = 164.5, n=257). On the contrary, the influence of peer attitude toward appearance on ED risk had the least magnitude of difference.

**Table 4:** The differences between mean scores of Sociocultural Attitudes Toward Appearance Questionnaire-4 (SATAQ-4) among eating disorders risk P-value < 0.05 is statistically significant.

SATAQ-4	Eating Disorders Risk	N	Mean Rank	Mann-Whitney	p-value
Internalization of thinness	Not at risk	257	164.5	-7.06	< .001
	At risk	127	249.2		
Internalization of masculinity	Not at risk	257	170.2	-5.62	< .001
	At risk	127	237.6		
Family attitude toward appearance	Not at risk	257	168.0	-6.17	< .001
	At risk	127	242.0		
Peer attitude toward appearance	Not at risk	257	171.4	-5.36	< .001
	At risk	127	235.1		
Media attitude toward appearance	Not at risk	257	169.6	-5.79	< .001
	At risk	127	238.9		
Total score on SATAQ-4	Not at risk	257	158.8	-8.46	< .001
	At risk	127	260.7		

**Figure 3:**
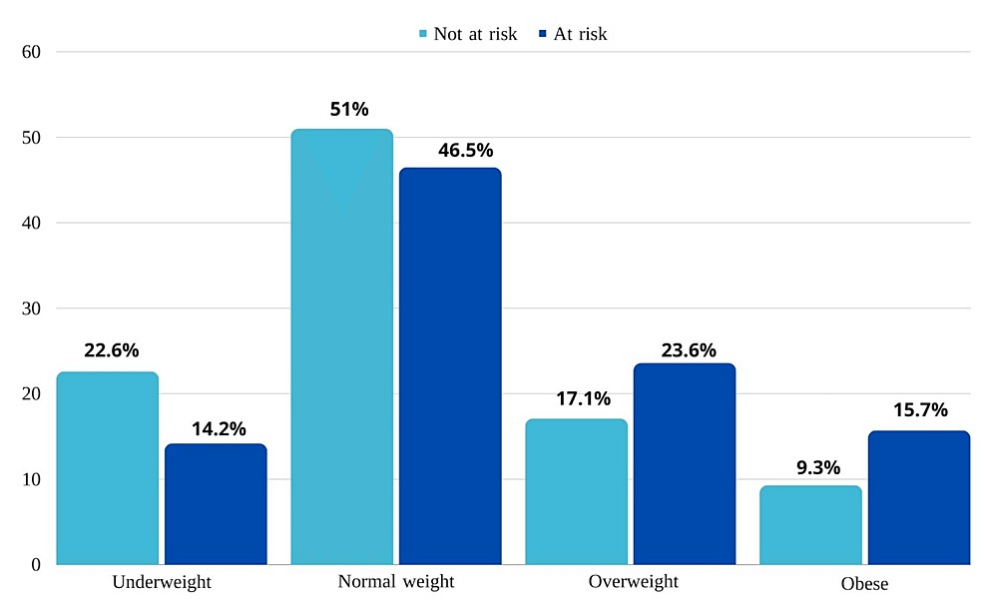
Eating disorder risk among BMI

**Figure 4:**
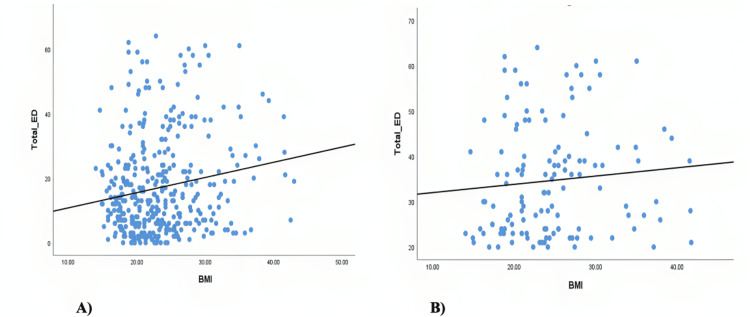
Relationship between Eating Attitudes Test scores and BMI (N=384) A) Relationship between Eating Attitudes Test scores and BMI in all participants (n=384). B) Relationship between Eating Attitudes Test scores and BMI for ED risk sample participants. BMI = body mass index, ED = eating disorder

Figure 3 illustrates the ED risk of participants' BMI; females who were obese demonstrated the greatest prevalence of ED risk, as the majority of obese participants (20 out of 44 participants) were at risk of ED. Additionally, 23.6% (n=30) of participants who were overweight were at risk of ED. And 46.5% (n=59) of participants who had normal weight were at risk of ED. Females who were underweight had the lowest prevalence of ED risk at 14.2% (n=18). In addition, there was a considerably weak positive correspondence between eating attitudes test scores and BMI, r (382) = .172, p = .001. There was no relationship between EAT scores and BMI for the ED risk sample, r (125) = .143, p = .109, as evident in Figure 4.

Table 5 shows a comparison between idealized standards of body weight and normal BMI (18.5-24.9%). A Wilcoxon signed-ranks test indicated the difference between idealized standards of body weight and normal BMI, p =.001 for the whole study sample, and likewise for no ED risk sample.

**Table 5:** Comparison between idealized standards of body weight in society and normal BMI * P-value < 0.05 is statistically significant.

Sample	Idealized Standards of Body Weight in Society Mean (SD)	Normal BMI Mean (SD)	P-value
All sample (n =384)	20.7 (2.5)	23.3 (5.5)	< .001*
Eating disorders (ED) no risk sample (N = 257)	20.6 (2.8)	24.5 (6.2)	< .001*
Eating disorders (ED) risk sample (N= 127)	20.7 (2.4)	22.6 (4.9)	< .001*

## Discussion

The objective of this paper is to explore the association of certain sociocultural attitudes on developing ED among young females in Almadinah Almunwarrah, Saudi Arabia.

This study has found that approximately 57% (n=218) of the participants in this study were from 18 to 22 years old, and 33.1% (n=127) of the participants scored 20 or more on the EAT-26 scale and were considered at risk for ED. The percentage of individuals who have a higher probability of developing an ED was one-third of the participants which is relatively similar to another study done on students at the University of Sharjah UAE [6].

Women of younger age groups are more vulnerable to being under the influence of sociocultural attitudes, thus they are more susceptible to developing risky eating behaviors [2]. One of the many causes is that at this age, young women start to develop an idea of their self-representation under the effect of family pressure, social media pressure, and peer pressure [6].

In this study, family pressure regarding body shape and weight showed a higher incidence in participants who were more at risk of developing ED (P﹤0.001). Similar results have been observed in another study done on female students in Jeddah where 45% (n=226) of the participants expressed family pressure to lose weight [10] and 50% (n=328) in another study done on female students in Dammam [11]. There are different factors relating to the effect of family on developing disordered eating behaviors, these include being labeled by family members as “overweight” earlier in life [12] and having mothers who exhibit disordered eating behavior themselves [13]. Furthermore, studies show a positive correlation between high education in both parents and grandparents and the risk of developing ED in their offspring [14,15].

Another factor that was observed was family income, as a study done by Hunger and Tomiyama suggests a strong positive association between family income and ED [12].

When discussing the source of peer pressure which is also a form of environmental influence, Dr Harris in a review of the development literature states that peer groups are one of the main factors that influence the development of disordered eating patterns. Pressure to fit in and meet the group norms is one of the most potent ways that peers can modify personality characteristics. This is not a direct obligation for friends to copy one another, but rather they subtly wish to share meaningful experiences with their peers that form part of their group identity [16].

Findings of other studies also indicated that peer interaction (e.g., discussing eating habits and dieting) and a desire for acceptance and popularity (i.e., believing that becoming thinner will make them more likable) both are factors of peer influence strongly associated with disordered eating. According to other studies, the significant association between peer pressure and disordered eating behaviors can be attributed to body comparison. Conversations between adolescents about appearance, weight, and dieting can implant the idea that the ideal beauty standard in the community is to be thin, which in turn can provoke the internalization of body image issues and dissatisfaction and lead adolescents to fall into unhealthy, disordered eating behaviors named successful by their peers to achieve that image [17].

To add to the review, the study that showed the influence of media and attitude toward appearance revealed a high magnitude difference between females who are at risk of developing ED and those who weren’t (P<0.001). And as shown in Figure 2 influence came higher than family factors and peers respectively. These results seem to intensify the traditional belief that exposure to media that advertise the thin bodies of social-media influencers triggers symptoms of ED among women who feel insecure about their bodies [18]. Another study proposes that women are more influenced by media due to the usual depiction of femininity with thin, slim bodies and virtues that are appealing to society like self-discipline, assertiveness and wealth, with what defies the norm or opposes these virtues being illustrated as ugly and unattractive [18]. Another study suggested that having a high BMI and being in a society that values a thin body which is influenced by media exposure is likely to increase the self-relevance of thinness and body dissatisfaction [19]. On the other hand, a study that was conducted in Kuwait states that the dissatisfaction with body image that came from media in Eastern society is initially adopted from Western media and Western lifestyles [20].

The discrepancy between actual body image (the current physical size as perceived) and ideal body image (desired body size) is frequently used as a measure of body dissatisfaction [21,22].

Most frequently, this discrepancy consists of desiring a thinner body. This study showed a difference in the idealized body weight and what is considered a normal BMI across the whole sample, regardless of being at risk of developing ED or not which indicates bodily dissatisfaction in the whole sample in general, but as Figure 3 draws conclusion that a higher BMI can lead to a higher risk of ED and that might be true as we observe from the figure that people who are considered obese had the highest ED risk. So, to confirm a comparison was tested to find the relationship between the BMI and score of EAT-26, and the results in Figure 4A of the whole sample 384 showed a significant weak positive correlation between BMI and risk of developing an ED. And the people who were reported to be at risk of ED were also measured in Figure 4B and showed no correlation between BMI and developing risk of ED. 

A wide-ranging literature provides evidence that this desire for a thinner body is generalized particularly among women [20]. And that the desire for underweight BMI is caused by viewing thin bodies as the ideal [23].

This mindset was evident in our study as it showed a great difference between what was viewed as “ideal” or “desired” body weight and what normal BMI actually is. Similar outcomes were seen in a previous study measuring females' understanding of normal BMI, especially in relation to their own idealized bodies, to determine whether women intentionally glorify bodies classified as “underweight"[23].

The aim of the current review was to gain insight if women were aware that they are idealizing underweight bodies, or if they do so unintentionally because they have an incorrect perception of what a categorically "normal" weight BMI looks like. Participants frequently incorrectly predicted the bodies' BMIs, however, they did so to a larger extent when they viewed bodies as an extension of their own, i.e., following the figure rating scale task. These results imply that women have inaccurate conceptions of the ideal body size, and that they frequently have greater misperceptions of the bodies of those around them, which may be causing people to idealize underweight bodies [23].

The goal of this study was to look deeper into the association of certain sociocultural attitudes on developing ED among females in Almadinah Almunwarrah, and this study has found that approximately 33.1% (n=127) of the participants scored 20 or more on the EAT-26 scale and were considered at risk for ED. Regarding risk factors of ED, we discussed media attitudes toward appearance, followed by family pressure regarding body shape and weight, and then peer pressure which all were factors that played a huge part in developing ED at such a young age. The discrepancy between actual body image (the current physical size as perceived) and ideal body image (desired body size) is frequently used as a measure of body dissatisfaction [21,22].

This discrepancy is typically expressed as a desire for a slimmer body. This mindset was evident in our study as it showed a great difference between what was viewed as “ideal” or “desired” body weight and what body weight, and BMI actually are.

The main limitation of this study was that BMI calculations were based on self-reported measures of weight and height, which involved response bias that may have led to inaccuracy regarding recorded responses and results. The cross-sectional design of the study is another possible limitation as it displayed the association between risk factors without causation and so, the results cannot be generalized. For future research, we recommend the inclusion of in-person interviews and height-weight documentation to limit the possibility of bias regarding data collection.

## Conclusions

In conclusion, this study highlights the role of sociocultural attitudes on the development of ED among young females in Almadinah Almunawarah, Saudi Arabia. Results show a high prevalence of risky eating behaviors, particularly among those who experience family and media pressure toward body shape and weight. Peer pressure was also identified as a significant risk factor. These findings emphasize the need for interventions that target sociocultural attitudes and provide support for vulnerable individuals. Education and awareness campaigns can play a crucial role in reducing harmful behaviors and promoting healthy body image. Further research is required to better understand the complex interplay between sociocultural factors and ED in this region.
